# Beta-3 Adrenoceptor Agonism Protects the Enteric Nervous Tissue Against Hyperoxia-Induced Damage

**DOI:** 10.3390/cells14070475

**Published:** 2025-03-21

**Authors:** Patrizia Nardini, Luca Filippi, Virginia Zizi, Marta Molino, Camilla Fazi, Matteo Chivetti, Alessandro Pini

**Affiliations:** 1 Department of Experimental and Clinical Medicine, University of Florence, 50139 Florence, Italy; patrizia.nardini@unifi.it (P.N.); virginia.zizi@unifi.it (V.Z.); marta.molino@unifi.it (M.M.); matteo.chivetti@edu.unifi.it (M.C.); 2 Neonatology and Neonatal Intensive Care Unit, Department of Clinical and Experimental Medicine, University of Pisa, 56124 Pisa, Italy; luca.filippi@unipi.it; 3 Department of Pediatric, Meyer Children’s University Hospital, 50139 Florence, Italy; camilla.fazi@unifi.it

**Keywords:** BRL37344, beta-3 adrenoceptor, beta-3 adrenoceptor agonism, development, hyperoxia, ileum, nervous tissue, oxidative stress, oxygen, ENS

## Abstract

The beta-3 adrenergic receptor (β3-AR), whose expression is modulated by oxygen levels, was found to play a key role in organ maturation, and its agonism was reported to mitigate hyperoxia-induced large bowel damage by preventing organ hypoplasia, preserving epithelial integrity, vascularization, and the neurochemical coding in the colonic myenteric plexus. This study explored the effects of β3-AR agonism in preventing hyperoxia-related alterations on the ileal enteric nervous system (ENS). Sprague–Dawley rat pups were reared under normoxia or hyperoxia (85%) during the first two weeks after birth and treated or not with the β3-AR agonist BRL37344 at 1, 3, or 6 mg/kg. Hyperoxia caused an imbalance of inhibitory nitrergic and excitatory cholinergic neurons in both the myenteric and submucosal plexuses and decreased the amounts of neurons in the submucosal plexus and that of S100β^+^ and GFAP^+^ glial cells in the myenteric plexus. Administration of 3 mg/kg BRL37344 preserved the neuronal chemical coding and partially prevented the loss of myenteric GFAP^+^ glial cells, while it did not counteract submucosal neuronal loss. Our findings indicate the potential of β3-AR agonism as a new therapeutic strategy for hyperoxia-induced ileal ENS alterations.

## 1. Introduction

The beta-3 adrenergic receptor (β3-AR) was first identified in the adipose tissue in 1989 [1], where it plays an essential role in regulating lipolysis and thermogenesis [2]. Since then, research has focused increasingly on the function of β3-AR across various organs under physiological and pathological conditions, mainly due to its distinctive regulatory mechanisms. Unlike other beta-adrenoceptor subtypes, β3-AR demonstrates low sensitivity to natural ligands (catecholamines) [3], suggesting its potential involvement in non-canonical signaling pathways in physiological and pathological conditions. Additionally, it exhibits resistance to desensitization, attributed to the absence of a consensus sequence for protein kinase A [3], making it a promising candidate for long-term pharmacological treatment. Moreover, β3-AR is highly responsive to oxygen levels, showing high expression during hypoxic intra-uterine life, followed by a swift decrease immediately after birth [4]. This latter characteristic has prompted investigations into its role in organ development and maturation. Considering that activation of β3-AR has also been demonstrated to protect against the harmful effects of reactive oxygen species (ROS) [5,6], its possible involvement in treating conditions related to prematurity, which is characterized by elevated oxidative stress due to anticipated exposure to normoxia [7], has become increasingly compelling. In this regard, β3-AR agonism has been demonstrated to prevent pathological retinal vascularization in an animal model of retinopathy [8]. Recent studies from our group on an in vivo model of rat neonatal hyperoxia-induced damage indicate that this receptor plays a key role in intestinal maturation and that its activation mitigates the adverse effects of elevated oxygen levels. In particular, β3-AR agonism improves the maturation of the colon by reducing oxidative stress, preventing organ hypoplasia, promoting mucin production, and maintaining the correct neurotransmitter coding by the myenteric plexus neurons [9]. Additionally, β3-AR agonism was also found to exert protection of the ileum by maintaining epithelial integrity and mucin production and promoting vascularization [10]. However, this latter investigation only focused on the ileal mucosa and its vasculature, whereas the potential effects of β3-AR agonism on the ileal enteric nervous system (ENS), which can have significant functional relevance, remain unexplored.

To address this gap, we have carried out the present study to investigate whether BRL37344, a widely used β3-AR agonist, could protect the ileal ENS using the same well-assessed model of rat neonatal hyperoxia-induced damage.

## 2. Materials and Methods

### 2.1. Experimental Model and Drug Administration

Pregnant Sprague-Dawley rats (Crl:CD(SD)) were individually housed in controlled environments, maintaining stable temperature conditions at 25 ± 2 °C. These rats were subjected to 12-h light and dark cycles, with unlimited access to food and water. Following delivery, the pups were pooled and randomly assigned to treatments in either ambient oxygen levels (normoxia, 21%) or oxygen-enriched environment (hyperoxia, 85%). Hyperoxia was sustained within an animal chamber (BioSpherix, Parish, NY, USA), with oxygen levels continuously monitored using a ProOx P110 probe (BioSpherix). The treated groups received subcutaneous injections of the selective β3-AR agonist BRL37344 (Merck, Darmstadt, Germany), administered every 12 h from birth (P0) until day 14 (P14). Every 24 h, the lactating dams were rotated between normoxia and hyperoxia to prevent high oxygen-related toxicity.

The neonatal rats were divided into six experimental groups: (1) normoxia controls (pups reared in 21% oxygen and not treated); (2) normoxia + BRL37344 (3 mg/kg) (pups raised in 21% oxygen and treated with 3 mg/kg BRL37344); (3) hyperoxia controls (pups reared in 85% oxygen and not treated); (4) hyperoxia + BRL37344 (1 mg/kg) (pups exposed to 85% oxygen and administered with 1 mg/kg BRL37344); (5) hyperoxia + BRL37344 (3 mg/kg) (pups reared under 85% oxygen and treated with 3 mg/kg BRL37344); and (6) hyperoxia + BRL37344 (6 mg/kg) (pups reared in 85% oxygen and treated with 6 mg/kg BRL37344). Body weights were recorded daily before injections. At the end of the experiment, the animals were euthanized with sodium pentobarbital (800 mg/kg i.p.), and samples of the terminal ileum were collected 3 cm from the cecum. Samples were subsequently washed in 0.1 M phosphate-buffered saline (PBS) at pH 7.4 and processed for morphological evaluations.

### 2.2. Sample Collection and Process

Ileal samples were surgically removed as previously described and separated into three segments, each 1 cm long. The terminal and middle segments were utilized for paraffin-embedded cross-sections and whole mounts, respectively. The tissue was placed in a plate containing ice-cold PBS for the whole-mount preparation and longitudinally opened. The specimens were then pinned down, stretched on the black silicone-coated dish, and fixed at +4 °C with 4% paraformaldehyde for 2 h. The samples were subsequently stored in PBS with 0.1% sodium azide until the immunofluorescence evaluation. For the paraffin-embedded cross-sections, selected samples were fixed overnight at 4 °C in 4% PAF, dehydrated in ethanol, cleared in Bio-clear (Bio-Optica, Milan, Italy), paraffin-embedded, and sectioned to a thickness of 5 µm.

### 2.3. Immunofluorescent Analysis

The immunofluorescent evaluations were performed on both the whole-mount and cross-section preparations.

The myenteric whole-mount preparations were obtained by dissecting the mucosal and longitudinal muscle layers through dissection. Samples were blocked and permeabilized with a PBS-triton X (0.2% *v*/*v*)-bovine serum albumin (BSA, Applichem, Darmstadt, Germany; 3%, *m*/*v*) solution for 2 h at room temperature (RT). The primary antibody directed to glial fibrillary acidic protein (GFAP) (Table 1) was appropriately diluted in the PBS-triton X (0.1% *v*/*v*)-BSA (1.5% *m*/*v*) solution and incubated overnight (ON) at +4 °C. The samples were rinsed 3× (20 min) with PBS under gentle agitation; then, the appropriate Alexa Fluor 488-conjugated secondary antibodies (Jackson ImmunoResearch, Ely, UK), diluted in the PBS-triton X (0.1% *v*/*v*)-BSA (1.5% *m*/*v*) solution, were applied for 2 h at RT. After three final washes in PBS (20 min), nuclei were stained with Hoechst solution (1:1000; Merck, Darmstadt, Germany), and the specimens were mounted with an anti-fade mounting medium (Mount Quick aqueous, Bio-Optica, Milan, Italy).

The GFAP immunostaining was visualized and acquired with a Leica Stellaris 5 confocal microscope (Leica Microsystems, Milan, Italy) equipped with a 20× objective with fixed laser intensity. Confocal images were acquired at high resolution (1024 × 1024 pixels × 10 µm), combining the mosaic mode to evaluate the entire whole-mount segment area and 1 µm z-stack mode to scan a total thickness of 10 µm per sample. Fiji-3 and Java 8 software (NIH) were used to segment the z-stack maximum intensity projections. Only the intra-ganglionic GFAP-immunolabeled area was considered in the morphometric analysis. In detail, for each specimen, at least 20 ganglia were identified and manually selected as regions of interest (ROIs); here, the GFAP positive area was thresholded and quantified. The results were expressed as the ratio of the GFAP^+^ area over the total analyzed area.

After rehydration, the ileal cross-sections were rinsed in sodium citrate buffer-Tween (0.05% *v*/*v*) (10 mM, pH 6) for 10 min at 90–92 °C for antigen retrieval. After blocking with 1.5% BSA to reduce non-specific binding, the sections were incubated ON at 4 °C with the primary antibodies (Table 1). For the double immunofluorescence, the sections were first incubated ON with a solution containing the two primary antibodies diluted adequately in PBS with 1% BSA and then for 2 h at RT with fluoro-chrome-conjugated secondary antibodies. The sections were mounted in an aqueous anti-fade medium (FluoroshieldTM with DAPI, Thermo Fisher Scientific, Waltham, MA, USA). Negative control was obtained by omitting the primary antibody. The immunolabeled cross-sections were observed under the Leica Stellaris 5 confocal microscope equipped with 20× or 63× PlanApo objectives. The total numbers of cells positive to HuC/D-, neuronal nitric oxide synthase (nNOS)-, choline acetyltransferase (ChAT)-, SRY-Box transcription factor (SOX)10-, and S100 calcium-binding protein (S100)β found within the myenteric and submucosal ganglia throughout the entire section were evaluated by two independent observers (P.N. and V.Z.) who were blinded to the experimental groups. Results are the percentage of nNOS^+^- or ChAT^+^-cells over the HuC/D^+^-cells per section, expressed as the mean ± S.D. of at least three sections per animal.

### 2.4. Statistical Analysis

Data are expressed as the mean ± standard deviation (S.D.) from at least 11 animals per group. Statistical analysis was conducted using a one-way ANOVA, followed by Tukey’s multiple comparison test. All statistical analyses were conducted using GraphPad Prism 9.0 software (GraphPad Software, San Diego, CA, USA).

## 3. Results

### 3.1. β3-AR Expression on the ENS

**Figure 1:**
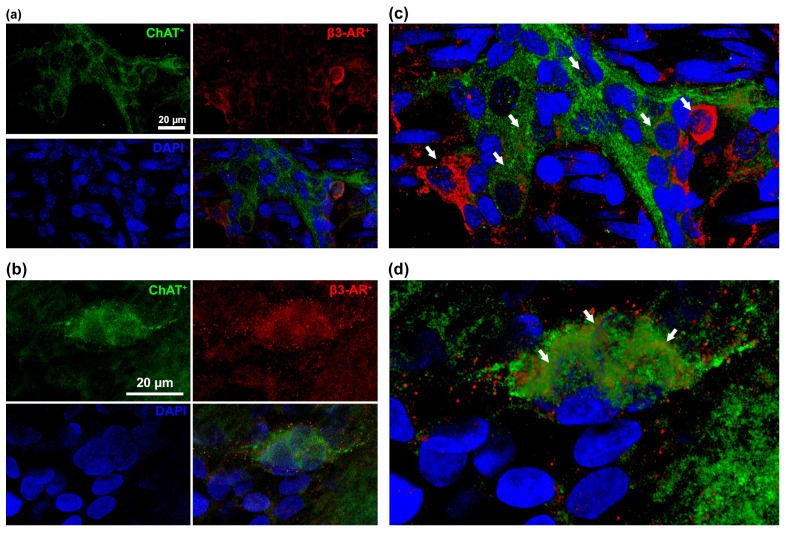
β3-AR-expressing cell types of the ileal ENS. Representative images of β3-AR^+^ (red) and ChAT^+^ (green) neurons in ileal myenteric (**a**) and submucosal (**b**) plexus. (**c**,**d**) 3D deconvolutions. White arrows highlighted the co-expression of the β3-AR+ and ChAT+ proteins. Nuclei are counterstained blue with DAPI. Scale bar = 20 µm.

To evaluate whether β3-AR agonism could protect the ENS against hyperoxia-induced damage, we first identified the specific cell types expressing the β3-AR. The immunofluorescent analysis revealed that β3-AR was expressed on ChAT^+^ neurons of both the ileal myenteric and submucosal plexuses (Figure 1). This finding is in keeping with the previous observations of a selective localization of β3-AR in a neuronal ChAT^+^ subpopulation in the colon [9,11,12].

### 3.2. Protective Effect of BRL37344 on the Hyperoxia-Exposed Myenteric Plexus

**Figure 2:**
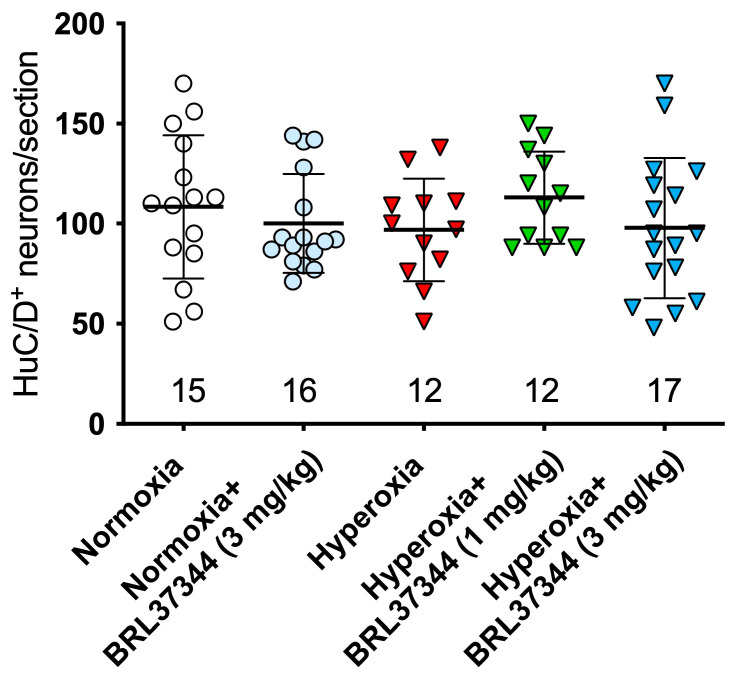
The pan-neuronal marker HuC/D was utilized to identify the neuron number per section (n. at the bottom) within the myenteric ganglia. No statistically significant differences were observed among the experimental groups. Values are expressed as mean ± S.D.

The adverse effects of hyperoxia exposure on the ileal myenteric plexus were first evaluated by counting the neurons expressing the pan-neuronal marker HuC/D. This analysis revealed no significant differences in the total neuronal number among the noted experimental groups (Figure 2). This finding is consistent with our previous study on the colonic myenteric plexus using the same rat neonatal hyperoxia-induced damage model [9]. Of note, the highest BRL37344 dose (6 mg/kg) resulted in the death of all the hyperoxia-exposed rats within a few days after birth, thus impeding any assessment of the considered markers.

**Figure 3:**
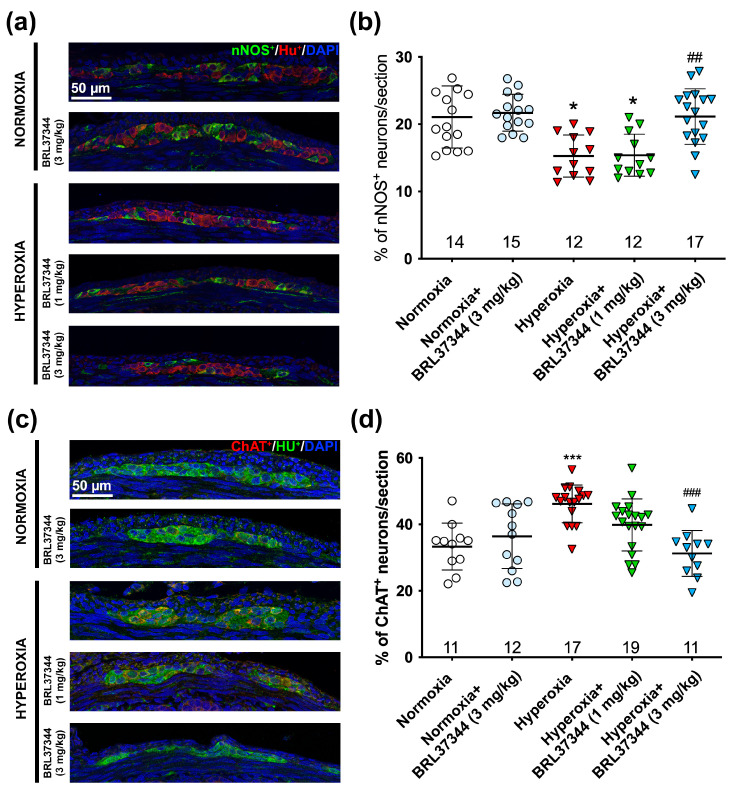
Immunolabeling and quantification of nNOS and HuC/D and ChAT^+^/HuC/D^+^ in the myenteric ganglia. Representative images depict nNOS^+^ (green) and HuC/D^+^ (red) neurons (**a**) and ChAT^+^ (red) and HuC/D^+^ (green) neurons (**c**), with nuclei counterstained blue with DAPI. Analysis of the percentage of nNOS^+^/HuC/D^+^ neurons (**b**) and ChAT^+^/HuC/D^+^ neurons (**d**) revealed that hyperoxia induced a statistically significant decrease in the nitrergic neuronal populations and increase in the cholinergic ones. Administration of 3 mg/kg BRL37344 effectively prevented these alterations. No significant differences were revealed between the rats treated with or not with the β3-AR agonist under normoxia. Values are expressed as mean ± S.D. * *p* < 0.05 and *** *p* < 0.001 vs. normoxia; ## *p* < 0.01 and ### *p* < 0.001 vs. hyperoxia. Scale bar = 50 µm.

While no differences were detected in the overall number of neurons, hyperoxia did cause relevant changes in their chemical coding. We performed nNOS+ and ChAT^+^ neuron counts and calculated the percentages of nNOS^+^/HuC/D^+^ and ChAT^+^/HuC/D^+^ to explore this aspect further. Compared to the control pups raised in normoxic conditions, those exposed to hyperoxia exhibited a statistically significant reduction in nNOS^+^ neurons and an increase in ChAT^+^ neurons (Figure 3a–d). Administration of 3 mg/kg BRL37344 effectively preserved both the nitrergic and the cholinergic neuronal subpopulations against the changes induced by excess oxygen (Figure 3a–d). In contrast, 1 mg/kg BRL37344 was not effective (Figure 3a–d). No differences were revealed between the control and BRL37344-treated animals raised under normoxic conditions (Figure 3a–d).

**Figure 4:**
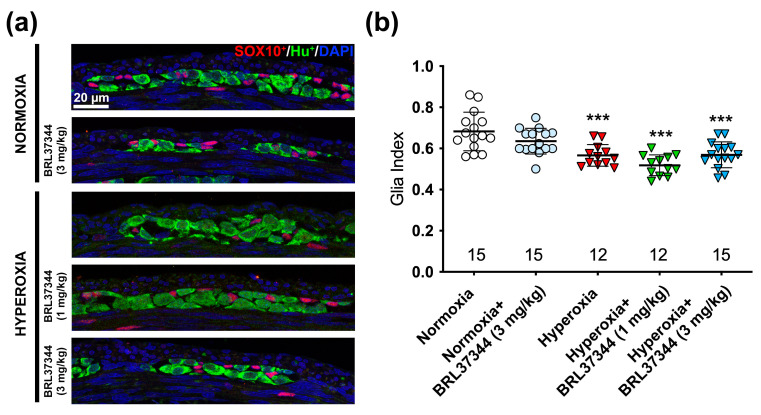
Immunolabeling and quantification of glial SOX10^+^ and neuronal HuC/D^+^ cells in the myenteric ganglia. (**a**) Representative images of SOX10^+^ (red) and HuC/D^+^ (green) cells, with nuclei counterstained blue with DAPI. (**b**) Analysis of SOX10^+^ to HuC/D^+^ cell ratio revealed that hyperoxia significantly decreased the glial cell number. Administration of BRL37344 at both doses did not prevent this alteration. No significant differences were observed in the normoxic rats treated or not with the β3-AR agonist. Values are expressed as mean ± S.D. *** *p* < 0.001 vs. normoxia. Scale bar = 20 µm.

Our findings revealed that the β3-AR was only expressed by ChAT^+^ neurons and not by glial cells, suggesting that the pharmacological targeting of this receptor did not directly affect glial cells. However, it has been established that a cross-talk between enteric neurons and glial cells is essential for the proper functionality of enteric neuronal circuits, and alterations of such interactions are associated with intestinal dysfunction [13]. Therefore, we extended our investigations to glial cells by counting SOX10^+^ cells and calculated the glia index as the ratio between SOX10^+^ and HuC/D^+^ cells, excluding those that co-expressed both markers, as they may be undifferentiated neuronal precursors [14]. As reported in Figure 4, hyperoxia exposure significantly reduced the glia index compared to the normoxic control, but the treatment with BRL37344 at both doses had no beneficial effects. Although the overall neuron count of neurons remained unchanged, we observed a marked reduction in the glia index among the hyperoxia-exposed pups. This finding led us to delve deeper into the principal glial markers S100β^+^ and GFAP^+^ affected by the elevated oxygen levels.

**Figure 5:**
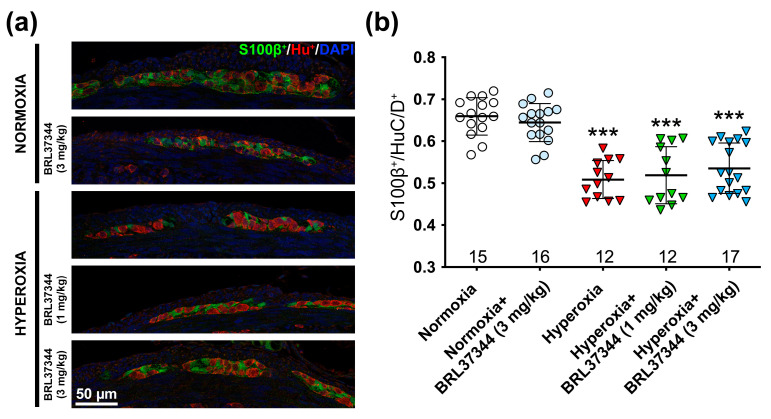
Immunolabeling and quantification of S100β^+^ glial and HuC/D^+^ neuronal cells in the myenteric ganglia. (**a**) Representative images of S100β^+^ (green) and HuC/D^+^ (red) cells, with nuclei counterstained blue with DAPI. (**b**) Morphometric analysis showed fewer S100β^+^ glial cells in all the pups raised in hyperoxia. Administration of BRL37344 at both doses did not prevent this alteration. No significant differences were observed in the normoxic rats treated or not with the β3-AR agonist. Values are expressed as mean ± S.D. *** *p* < 0.001 vs. normoxia. Scale bar = 50 µm.

We determined the ratio of S100β^+^ to HuC/D^+^ cells, excluding those that co-expressed both markers, as it has been reported that numerous immature HuC/D^+^ neurons in the adolescent rodent gut also express S100β [14]. As shown in Figure 5, hyperoxia exposure significantly decreased the S100β^+^ glial subpopulation compared to the normoxic controls. Administration of BRL37344 at both doses had no beneficial effects.

**Figure 6:**
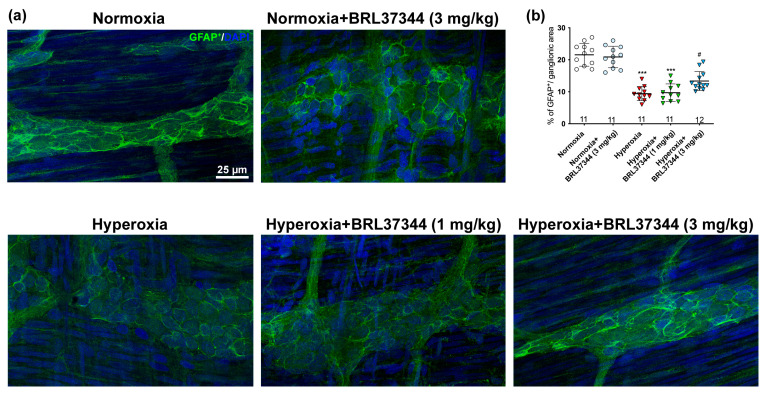
Immunolabeling and quantification of GFAP in the myenteric ganglia whole mount preparations. (**a**) Representative images and (**b**) relative morphometric analysis of GFAP positive area (green). Nuclei are counterstained blue with DAPI. Compared with the normoxic controls, the pups reared under hyperoxia showed a reduced GFAP^+^ area. BRL37344 had a partially protective effect at 3 mg/kg but not at 1 mg/kg. Values are expressed as mean ± S.D. *** *p* < 0.001 vs. normoxia; # *p* < 0.01 vs. hyperoxia. Scale bar = 25 µm.

A morphometric analysis of the whole mount preparations was carried out to evaluate the impact of hyperoxia on the myenteric GFAP^+^ expression. The pups exposed to hyperoxia showed a significant reduction of the overall GFAP immunofluorescent area compared to the normoxic controls (Figure 6a,b). Administration of 3 mg/kg BRL37344 had a partial protective effect but did not fully restore the GFAP expression to levels of the normoxic controls (Figure 6a,b). In contrast, 1 mg/kg BRL37344 did not prevent this alteration (Figure 6a,b). No differences in GFAP immunoreactivity were observed among the rats treated with the β3-AR agonist under normoxic conditions compared to the untreated rats.

### 3.3. Protective Effect of BRL37344 on the Submucosal Plexus

**Figure 7:**
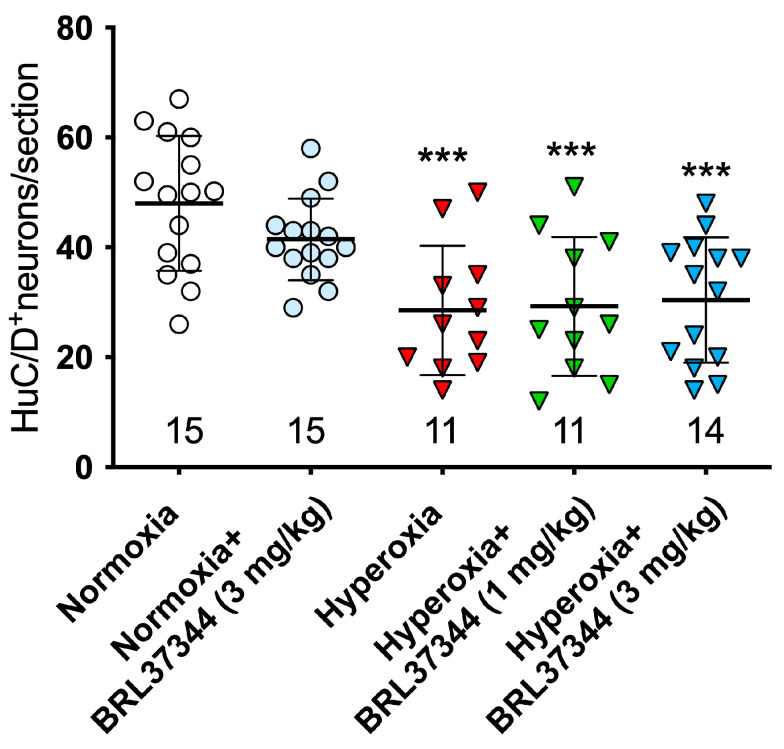
Total number of HuC/D+ neurons per section (n. at the bottom.) in the ganglia of the ileal submucosal plexus. All the pups reared under hyperoxia showed a significantly lower number of neurons than the normoxic controls. No significant differences were observed upon administration of BRL37344 at both doses. Values are expressed as mean ± S.D. *** *p* < 0.001 vs. normoxia.

To investigate the adverse effects of high oxygen levels on the submucosal plexus, we carried out morphometric analyses similar to those performed on the myenteric ganglia. We first assessed the total number of HuC/D^+^ neurons, and we found that the pups raised under hyperoxia showed fewer neurons than their normoxic counterparts. At the same time, BRL37344 at both doses had no significant protective effects (Figure 7).

**Figure 8:**
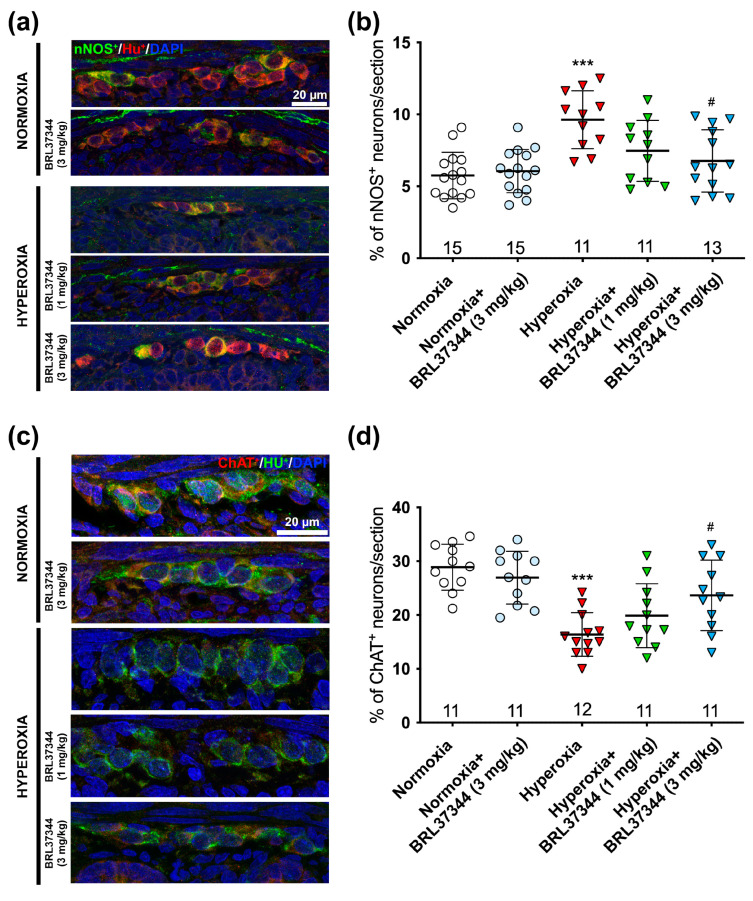
Immunolabeling and quantification of nNOS^+^/HuC/D^+^ and ChAT^+^/HuC/D^+^ neurons in the submucosal plexus. Representative images showed nNOS^+^ (green) and HuC/D^+^ (red) neurons (**a**) and ChAT^+^ (red) and HuC/D^+^ (green) neurons (**c**), with nuclei counterstained blue with DAPI. Analysis of the amount of the nNOS^+^/HuC/D^+^ neurons (**b**) and ChAT^+^/HuC/D^+^ neurons (**d**) revealed a statistically significant increase in the nitrergic neural subpopulation and a decrease in the cholinergic one in the hyperoxia-exposed pups compared to those reared in normoxia. Treatment with 3 mg/kg BRL37344, but not 1 mg/kg, effectively counteracted these alterations. No significant differences were observed in the normoxic rats treated or not with BRL37344. Values are expressed as mean ± S.D. *** *p* < 0.001 vs. normoxia; # *p* < 0.05 vs. hyperoxia. Scale bar = 20 µm.

Next, we evaluated the impact of hyperoxia exposure on the chemical coding of the submucosal plexus neurons by analyzing the ratios of nNOS^+^ to HuC/D^+^ and ChAT^+^ to HuC/D^+^. In contrast to the myenteric plexus, excess oxygen increased the number of nNOS^+^ neurons while it decreased that of ChAT^+^ ones (Figure 8a–d). These alterations were completely prevented by the 3 mg/kg BRL37344 administration, whereas the 1 mg/kg dose was not effective (Figure 8a–d). As expected, no differences were observed between the normoxic controls treated or not with BRL37344 (Figure 8a–d).

**Figure 9:**
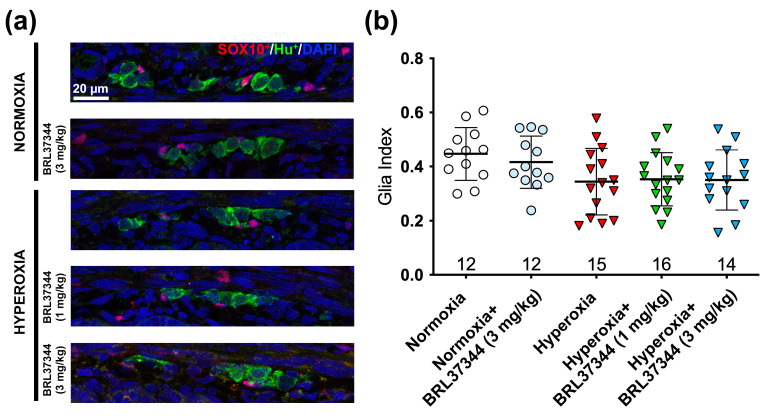
Immunolabeling and quantification of SOX10^+^ glial cells and HuC/D^+^ neurons in the submucosal ganglia. (**a**) Representative images of SOX10^+^ (red) and HuC/D^+^ (green) cells, with nuclei counterstained blue with DAPI. (**b**) Analysis of the SOX10^+^/HuC/D^+^ cell ratio revealed no significant differences among the experimental groups. Values are expressed as mean ± S.D. Scale bar = 20 µm.

Following the experiments on the myenteric plexus, we evaluated the glia index in the submucosal plexus by the SOX10^+^/HuC/D^+^ cell ratio. This analysis revealed a slight, non-significant reduction of glial cells in all the hyperoxic groups (Figure 9). Given this result, we did not further analyze the other glial markers in the submucosal plexus.

## 4. Discussion

It is widely recognized that neonatal hyperoxia exposure impacts intestinal maturation. However, most existing research has focused primarily on the adverse effects of excess oxygen on mucosal morphology, mucin production, and vascularization, leaving the influence on the ENS largely unexplored [9,10,15,16]. In this context, our previous studies have demonstrated that hyperoxia induced alterations in the neuronal chemical coding in the colonic myenteric plexus [10], while no data exist on the effects of excess oxygen on the glial and neuronal components of the ileal ENS, which plays a pivotal functional role in nutrient absorption. To bridge this knowledge gap, in the present study, we first describe the alterations induced by the exposure of newborn rats to hyperoxia on the ileal ENS, using the same well-characterized in vivo model exploited for the previous investigation [9,10], and we highlight the protective effects of the selective β3-AR agonist BRL37344.

The pharmacological targeting of β3-AR was achieved using BRL37344, ((±)-(R*,R*)-[4-[2-[[2-(3-Chlorophenyl)-2-hydroxyethyl]amino]propyl]phenoxy]acetic acid sodium hydrate), a compound that has shown high selectivity for β3-AR, with a Ki of 430, compared to the other β-ARs, exhibiting Ki values of 37,900 and 9170 for β1-AR and β2-AR, respectively, in rodents [3,17]. Its suitability for subcutaneous administration makes it more compatible with the neonatal rat model used in our study than Mirabegron, a second-generation highly selective β3-AR agonist, whose administration via oral gavage to newborn rats is particularly difficult. BRL37344 has been demonstrated to promote relaxation in various human tissues by activating nitric oxide synthase [18], large-conductance Ca(2+)-activated K(+) channels [19], or enhancing hydrogen sulfide production [20]. Specifically, it induced relaxation in the bladder [21], atrial myocardium [22], coronary micro-arteries [18], myometrium [23], and corpus cavernosum [20]. While BRL37344 has not received approval for human use, the insights gained from this compound were pivotal in highlighting the essential role of β3-ARs in human bladder relaxation, ultimately leading to the approval of mirabegron for treating overactive bladder in humans [19].

The first relevant finding of this study is that the cholinergic neurons in the myenteric and submucosal plexuses of the ileum express the β3-AR. This finding is in keeping with the previous observations that the β3-AR is present on the cholinergic neurons of the colonic myenteric plexus [9,11,24]. On the other hand, the lack of expression of this receptor by the enteric glial cells excludes a direct effect of its pharmacological targeting on these cells.

Second, this study demonstrates that hyperoxia significantly affected the neurochemical coding within the ileal myenteric plexus. In particular, we observed that excess oxygen increased the cholinergic neurons and reduced the nitrergic ones. The administration of BRL37344 (3 mg/kg) completely prevented these alterations. These results align with our previous research, which identified similar hyperoxia-induced changes in the myenteric plexus of the rat colon and a comparable protective effect of BRL37344, although in this case, β3-AR agonism only yielded partial protection. This discrepancy could be related to the different functions of the ileum and colon, as well as to differences in the degree of maturation of the gut tracts, which is known to proceed in cranial-to-caudal direction [25]. Moreover, our morphological analysis of the ileal cross-sections indicated that hyperoxia did not alter the total number of neurons per ganglion. It should be pointed out that this morphological approach only allows for counting cell numbers without providing insight into the overall structure of the ganglia, whose morphology could be altered by hyperoxia. To address this limitation, further experiments should be conducted using whole-mount preparations, which could offer a more comprehensive understanding of the overall structure of the plexus and enable more precise quantification of neuron numbers.

During postnatal gut development, the various enteric neuronal subtypes are still present in the neuronal plexuses, where they show significant plasticity in their reciprocal connections and with the enteric glia. This dynamic interplay influences the maturation of enteric neurocircuits [25]. In this context, we also explored the effects of hyperoxia on the glial population and assessed the impact of BRL37344 treatment. Despite the absence of β3-AR expression by these cells, our findings have shown that hyperoxia significantly reduced the glia index, decreasing both the S100β^+^ cells and the GFAP^+^ area. Of note, β3-AR agonist administration partially counteracted this decrease, preserving the expression of GFAP. The glia index in rodent ENS increases during gut maturation along with the acquisition of normal motility function, eventually reaching a value of 1.4 in adults [26]. This delayed, progressive increase in the number of glial cells can be explained by the fact that they typically differentiate after the enteric neurons [26]. In particular, S100β and GFAP expression progressively increases in the ENS within the first two weeks after birth and peaks at P36, when well-developed cell processes appear, indicating that a complete glial network has been formed similar to that of adults [26]. Glia is known to play crucial roles besides providing mechanical support to neurons; these include neuroprotection [27], intestinal motility regulation [28], and participation in the epithelial barrier [29]. The reduced expression of both glial markers induced by hyperoxia suggests that excess oxygen can negatively influence glial maturation and function, likely depleting glial precursors. Indeed, while there are currently no data on the effects of hyperoxia on enteric glia, it is well established that exposure to hyperoxia in neonatal stages results in the death of immature oligodendrocytes and delays their lineage maturation within the central nervous system [30]. The indirect protective effect of BRL37344 in preserving the GFAP expression strongly suggests its supportive role in ENS maturation, counteracting the toxic effect of hyperoxia and potentially helping to preserve ileal function. However, since most intra-ganglionic glial cells co-express S100β and GFAP [31], the current immunofluorescent analysis did not allow a clear identification of the specific glial subpopulation that benefited from the β3-AR agonist. Furthermore, the exact mechanism by which this treatment indirectly preserved glial GFAP expression is yet to be clarified. In this context, it appears that the well-known response of GFAP^+^ to proinflammatory stimuli [32] and its upregulation as a marker of enteric gliosis [33] are likely restricted to adult life rather than to perinatal ENS maturation.

Another relevant finding of this study is the marked difference in the response to hyperoxia between the submucosal and the myenteric plexus. At variance with the myenteric ganglia, excess oxygen significantly reduced the total neuron number within the submucosal plexus. Additionally, hyperoxia modified their chemical coding, increasing the nitrergic component and reducing the cholinergic neuron number, while no changes were observed in the glia index. The β3-AR agonist was able to preserve the correct neurochemical coding, but it was ineffective in counteracting the neuronal loss. Although it is well-known that neonatal exposure to hyperoxia leads to a significant decrease in the number of neuronal precursor cells (NPCs) in the central nervous system, which consequently diminishes the overall population of mature neurons [34], there are currently no data concerning the impact of hyperoxia on postnatal neurogenesis in the ENS. Under the same experimental conditions of hyperoxia exposure, we found no change in the number of neurons in the colonic [9] and ileal myenteric plexus, while we observed a decrease in neurons in the ileal submucosal plexus. Considering that in rodents, the development of the submucosal plexus follows that of the myenteric one and continues after birth in rodents [25], it is reasonable to speculate that excess oxygen may have hindered the postnatal submucosal neuronal maturation rather than causing cell death. However, further experiments focused on the NPC lineage retracement are needed to validate this hypothesis.

The ostensibly contradictory findings concerning the hyperoxia-induced alterations in submucosal chemical coding, which are opposite to those observed in the myenteric plexus, may be interpreted as a compensatory response. It is plausible that the hyperoxia-induced loss of the inhibitory nNOS neurons—known to be particularly susceptible to oxidative injury [35]—within the myenteric plexus could have led to enhanced contraction of the *muscolaris externa*, resulting in abnormally increased peristaltic movements. In this context, the decrease in excitatory cholinergic neurons and increase in inhibitory nitrergic ones in the submucosal plexus may be viewed as an adaptive response to counteract irregular segmentation and preserve the activity of the mucosa. Further morpho-functional ex vivo studies are necessary to validate this hypothesis, as the present neonatal rat model does not allow a direct assessment of feces or behavioral studies. The unexpected stability of the submucosal glia index upon hyperoxia exposure can be due to a reduced number of neurons or to the delayed maturation of the submucosal plexus compared to the myenteric plexus. If so, it is reasonable to hypothesize that additional changes could appear in a longer time.

Our study has some limitations. As previously reported, the pharmacological targeting of β3-AR was obtained using BRL37344 for its suitability for subcutaneous administration. This delivery method makes BRL37344 more compatible with the neonatal rat model than Mirabegron, whose administration via oral gavage to newborn rats is particularly difficult. However, BRL37344 has been demonstrated to act through β2-AR in limited tissues, such as skeletal muscle [36]. Consequently, it is not possible to completely exclude that some of the effects observed in our study may be due, at least in part, to BRL37344 agonism on β2-AR, which is known to be present on the submucosal VIP^+^ or calretinin+ neurons [37]. Nonetheless, a recent in vivo investigation into the role of β3-AR in the development of retinopathy of prematurity indicates that the effects of BRL37344 were specifically mediated by β3-AR, as demonstrated by its lack of effect when administered with SR59230A, a widely used β3-AR antagonist [8]. The second notable limitation of this study is the absence of any evaluation of the impact of hyperoxia and the potential beneficial effects of BRL37344 on the gut microbiota. This aspect is particularly significant, considering that postnatal gut development is crucial for establishing the gut microbiome [38]. However, such analysis would require different in vivo experimental approaches and more animals to achieve statistical significance. The final limitation of this study is the absence of functional analysis, which could enhance our understanding of the consequences of hyperoxia-induced changes in chemical coding, as well as the therapeutic potential of BRL37344 in gut dysmotility of prematurity. Such functional analysis requires the simultaneous assessment of various parameters—e.g., contractions of the circular and longitudinal muscles, intraluminal pressure, mucosal secretion, and lumen emptying [39]—which are outside the scope of the present experimental model.

Translating findings from animal research to humans requires caution due to significant differences in the ENS between species. For instance, the glia index in the human intestine is about 7-fold greater than in the mouse, suggesting a more relevant role for glial cells in humans than in rodents [40]. Nevertheless, our results indicate that β3-AR agonists can preserve the ileal ENS against hyperoxia-induced damage, likely favoring postnatal neuronal maturation. This finding may have important clinical implications for treating premature newborns, especially in mitigating the side effects of supplemental oxygen therapy.

## 5. Conclusions

In conclusion, this study demonstrates that neonatal exposure to excess oxygen significantly alters the ileal ENS, and that the selective β3-AR agonist BRL37344 at an appropriate dose (3 mg/kg) provides effective protection. These findings further highlight the potential of pharmacological targeting of the β3-AR as an innovative strategy for treating preterm infants, mitigating the many adverse effects of premature exposure to oxygen, especially when subjected to supplemental oxygen therapy. This potential therapeutic approach is particularly compelling considering that drugs such as mirabegron and vibegron, both β3-AR agonists, are already approved for treating overactive bladder [19]. These drugs are well tolerated and have a favorable safety profile, even in pediatric patients [41]. Our results may offer the scientific basis for repurposing mirabegron and vibegron, thereby expediting the translation of these findings from the bench to the bedside.

## Figures and Tables

**Table 1:** Primary and secondary antisera used in immunofluorescence analysis.

Antigen	Species	Source	Catalog Number	Concentration
B3-AR	Rabbit	GeneTex (Irvine, CA, USA)	GTX70685	1:300
ChAT	Chicken	GeneTex	GTX85450	1:200
GFAP	Chicken	Abcam (Cambrige, UK)	Ab4674	1:900
nNOS	Rabbit	Genetex	GTX133403	1:500
S100β	Rabbit	Genetex	GTX129573	1:500
Sox10	Rabbit	Abcam	Ab227680	1:50
HuC/D	Mouse	Invitrogen (Waltham, MA, USA)	A21271	1:200
**Secondary Antisera**				
Alexa Fluor 488	Donkey	Jackson ImmunoResearch (Ely, UK)	711-545-152	1:175
Alexa Fluor 488	Donkey	Jackson ImmunoResearch	703-545-155	1:175
Alexa Fluor 594	Donkey	Jackson ImmunoResearch	711-585-152	1:175
Alexa Fluor 594	Sheep	Jackson ImmunoResearch	515-585-062	1:175
Alexa Fluor 488	Sheep	Jackson ImmunoResearch	515-545-062	1:175
Alexa Fluor 594	Bovine	Jackson ImmunoResearch	805-585-180	1:175

## Data Availability

The datasets generated during and/or analyzed during the current study are available from the corresponding author upon reasonable request.
